# Investigating the Potential of Commercial-Grade Carbon Black-Filled TPU for the 3D Printing of Compressive Sensors

**DOI:** 10.3390/mi10010046

**Published:** 2019-01-10

**Authors:** Claudio Manganiello, David Naso, Francesco Cupertino, Orazio Fiume, Gianluca Percoco

**Affiliations:** 1 Politecnico di Bari, Dipartimento di Meccanica Matematica e Management, Viale Japigia 182, 70126 Bari, Italy; claudio.manganiello@tiscali.it (C.M.); oraziofiume@libero.it (O.F.); 2 Politecnico di Bari, Dipartimento di Ingegneria Elettrica e dell’Informazione, Via Re David 200, 70125 Bari, Italy; david.naso@poliba.it (D.N.); francesco.cupertino@poliba.it (F.C.)

**Keywords:** 3D printing, transducer, additive manufacturing, flexible, filament freeform fabrication, fused deposition modeling

## Abstract

The present research aims to exploit commercially available materials and machines to fabricate multilayer, topologically designed transducers, which can be embedded into mechanical devices, such as soft or rigid grippers. Preliminary tests on the possibility of fabricating 3D-printed transducers using a commercial conductive elastomeric filament, carbon black-filled thermoplastic polyurethane, are presented. The commercial carbon-filled thermoplastic polyurethane (TPU), analyzed in the present paper, has proven to be a candidate material for the production of 3D printed displacement sensors. Some limitations in fabricating the transducers from a 2.85 mm filament were found, and comparisons with 1.75 mm filaments should be conducted. Moreover, further research on the low repeatability at low displacements and the higher performance of the hollow structure, in terms of repeatability, must be carried out. To propose an approach that can very easily be reproduced, only commercial filaments are used.

## 1. Introduction

3D printing has the potential to deeply affect the manufacture of mechatronic devices, shaping highly complex objects and embedding external components into complex structures. The additive processes provide a cost-effective way of using self-developed and individually designed mechatronic devices, with a completely customizable design that can combine optical, chemical, electronic, electromagnetic, fluidic, thermal, and acoustic features, potentially at a very low cost. Even if the conductivity of materials is still a challenge, due to the blending of conductive materials in a polymer matrix, typically made of dielectric materials, very recently a wide variety of composite materials have become available, with interesting conductive properties. Unfortunately, these composites are often critical for 3D printing due to their thermal expansion coefficient, glass transition temperature, and mechanical properties. These drawbacks actually limit the diffusion of 3D printing for mechatronic devices, and research in this field is very recent and limited to highly customized materials and machines.

### Research Background

Traditionally fabricated sensors have some limitations, such as high production times, difficulty in producing elements with relatively small dimensions (micrometric features), difficulty in assembling micro components using imprecise and non-lasting fixing and/or gluing methods, high costs due to the miniaturization of the component, and poor large-scale reproducibility.

The additive manufacturing methods applied in the sensor industry seek to overcome some of these problems, guaranteeing lower production times and costs, with the possibility of embedding the transducers into structures (i.e., soft grippers) directly during the manufacturing.

In the current state of the art, additive manufacturing processes have proven to be an excellent alternative for the realization of customized sensors, while the traditional production methods are still the elective technology for high volume production.

3D printed sensors can be classified into three macro categories, including physical sensors, chemical sensors and biosensors [1].

The physical sensors are used to detect the variation of one physical quantity, caused by physical effects, through the variation of the electrical resistance or capacity.

In the literature, most 3D-printed sensors are built by exploiting the mechanical properties of the most common filaments used in extrusion-based additive manufacturing. Polylactic acid (PLA) and thermoplastic polyurethane (TPU) are among the most commonly used materials in additive manufacturing. These materials can be coupled with one conductive material, typically thermoplastics doped with carbon nanotubes, which can be extruded and deposited. As a reference, the study in [2] offers an overview of the commercially available electroconductive filaments, comparing the electrical features (resistance, conductivity and breaking point) to maximize the feasibility of industrial versions of the sensor and cross the threshold of large-scale production.

The use of an electroconductive ink is reported in [3], where an extensimeter is realized, with a coplanar structure of a few millimeters thickness, starting from a simple rectangularly shaped elastomeric base and depositing a coil-shaped feature made of an electroconductive ink, suitable for detecting elongation and torsion in laboratory experiments. Unfortunately, the deposition of electroconductive inks forces the two materials, with such different features, to be coupled.

In the literature, mainly bending, torque, and tensile sensors have been studied, while to the best of the authors’ knowledge, no 3D-printed compressive sensors have been proposed.

One example is [4], where a continuous conductive filament line is printed through a thin or hollow structure along the axis of the desired interaction. The bending sensors are therefore analog resistive sensors, where one end of the filament acts as an emitter, and the other, as a receiver. When the structure is bent, the carbon particles in the filament move away from each other. As a result, the resistance of the circuit increases proportionally.

The creation of 3D printed circuits is very interesting, and several solutions are proposed by some authors. Coupling and embedding extruded conductive materials into other materials is not a trivial task. A truly effective method for obtaining printed circuit production through additive manufacturing systems is not available yet, but it is self-evident that the potential advantages push research in this direction. Recently, a technique has been proposed for printing electronic flexural and capacitance sensors, thanks to a composite thermoplastic, as illustrated by [5].

The crucial point in the realization of sensors with purely additive technology is the interaction between the different materials (elastomeric base and electrically conductive material). In fact, taking the case of fused deposition modeling (FDM) printing, the superimposition of successive layers requires adhesion between the materials. Attention must be paid to the phenomenon known as cross contamination, that is, the possible local, partial bending of the materials, leading to loose conductive properties.

Concerning the typology of additive manufacturing processes, the reduced cost of the thermoplastic filaments and the attention they have received by the open source world have made filament freeform fabrication (FFF) the most widely used 3D printing technique, in spite of its low printing resolution and slow printing speed.

3D printing technology has been implemented either to completely produce sensors, or to carry out only a part of their production process, through the integration of different materials that embed active components [6].

In the literature, customized and complex solutions have been proposed, such as [7,8] (for an exhaustive review of 3D-printed sensors please refer to [9]).

Several patent applications relating to heterogeneous sensors in various fields have also been filed. One example is [7], which proposes a microsensor for electrophysiology and electrochemistry studies in vitro and in vivo for the detection of electroactive neurotransmitters. Another example is [8], which discloses a temperature sensor system based on a micro fluidic chip and a preparation method of the system.

Soft strain sensors are typically composed of a deformable conducting material patterned onto, attached to, or embedded in an inactive stretchable material, and this kind of sensor is eligible for 3D printing. On the market, conventional strain sensors are available. They are mainly piezoelectric sensors, often made of heterogeneous portions. However, the high standardization of manufacturing processes allows highly affordable sensors to be obtained.

However, such commercial sensors have substantially individual components, which need to be arranged mechanically in a structure or body under observation and be connected electrically to a measurement device.

When the miniaturization is particularly strong, the high affordability of individual commercial sensors is lost during their mechanical and electrical connection in defining a custom complex measurement system.

The possibility of manufacturing printed sensors allows the production of each individual sensor to be embedded together with a complex custom measurement structure, in which the electric wires are also embedded additively through the same manufacturing procedure. Thus, the main challenge is to exclusively use 3D printing by avoiding the implementation of heterogeneous components, which are often adopted to realize concentrated measurements. However, the affordability of printed sensors must be investigated, before future implementation, by bearing in mind that the sole degree of freedom is the shape conferred on the sensor itself.

In this context, the present research aims to evaluate the commercially available materials and machines for fabricating multilayer, topologically optimized transducers, which can be embedded into mechanical devices, such as soft grippers. Preliminary tests on the possibility of fabricating 3D-printed transducers using a commercial conductive elastomeric filament are presented. To the best of the authors’ knowledge, no sensor applications using such a product are described in the literature. On the other hand, the exploitation of commercially available materials and machines could dramatically affect the diffusion of 3D additive manufacturing of mechatronic micro components.

## 2. Materials and Methods

This paper reports an experimental study that aims to fabricate simple and low-cost 3D-printed strain sensors in the form of 3D-printed hollow parts, as potential compressive strain or tactile sensors, exploiting low-cost commercial equipment and materials. The FFF process (Ultimaker 3, equipped with the open source Software, Cura3.2, Ultimaker B.V., Geldermalsen, The Netherlands) has been selected, because of the wide availability of commercial filaments, such as the commercial conductive flexible filament, PI-ETPU 95-250 carbon black (thermoplastic polyurethane matrix with carbon black dispersion) by Palmiga Innovation ltd. (Jonstorp, Sweden), with the following features: Diameter: 2.85 ± 0.05 mm, Density: 1.3 g/cm^3^, Volume Resistivity: <800 Ω·cm.

**Figure 1:**
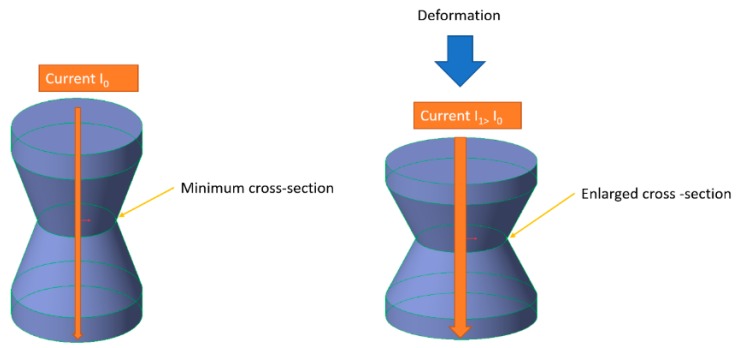
Principle of the 3D-printed transducer.

The experimental study analyzes the electrical properties of the parts, designed as an axisymmetric solid (an example is reported in Figure 1), but the results obtained can be extended to any other solid shape. A non-constant section with a narrow waist structure has been employed, which provides the possibility of modulating its mechanical and electrical properties, modifying the minimum section, but saving the additive manufacturability, without supports.

The working principle is shown in Figure 1. The axial symmetric PI-TPU 3D-printed workpieces are connected to an external electric circuit. If this solid is solicited by an axial force, it will be deformed with a height reduction and an increase in the minimum and average cross section. However, it is well known that FFF and 3D printing processes generally produce anisotropic solids, both macroscopically, due to the filament deposition in preferential directions, and microscopically, depending on the attitude of the filament to flow continuously and to adhere, layer by layer.

**Figure 2:**
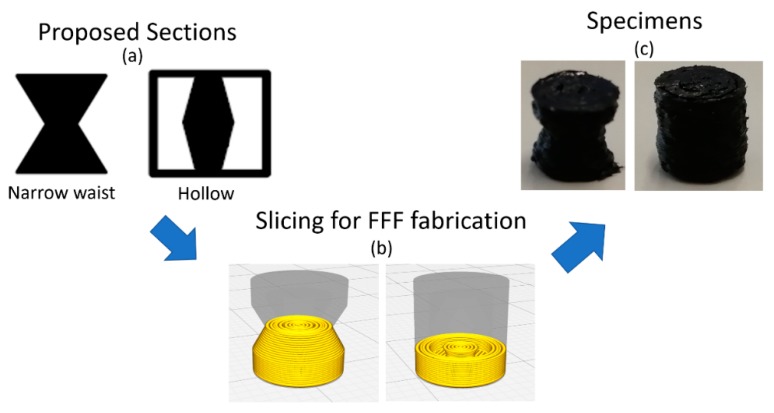
Principle of the 3D-printed transducer. (**a**) the sections analyzed; (**b**) the slicing stage; (**c**) the 3D printed specimens.

With the purpose of considering different stiffnesses of the transducer, two alternative shapes have been tested and compared with the solid transducer element. The authors selected the truncated cone as an elementary shape, symmetric solid, with a section area that is variable along the symmetry axis. This shape has been exploited following two approaches: a solid “narrow waist” structure and a hollow structure (Figure 2a).

**Figure 3:**
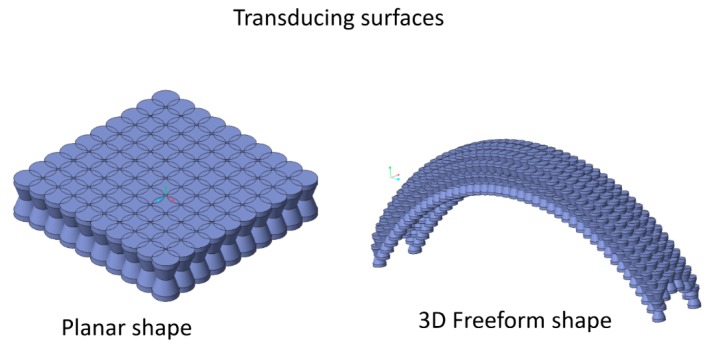
Possible parallel arrangements for extended transducing surfaces.

A more complex 3D transducer may consist of one or more elementary parts, arranged in a planar fashion or as the elementary cells of a 3D matrix to obtain a freeform shape (Figure 3), which can constitute a sensing surface that is embeddable into mechanical devices, such as robotic grippers, for instance.

The chosen size of the transducer was 7.5 mm, as the cylinder height, and 5 millimeters, as the base diameter. This size has been chosen, after several tests, as the best compromise between the smallest size and highest resolution of the 3D printer. This aspect is depicted in Figure 2c, where the fabricated elements are shown, evidencing the limitations of the material attitude, to be extruded in the Ultimaker 3.

The two considered geometries have been compared to a solid 3D printed cylinder. To make the test more reliable, three different samples have been printed for each type, using the same parameters on the 3D printer. The 3D printing of conductive TPU samples using the Ultimaker 3 proved to be challenging due to the features of the filament. The PI-ETPU 95-250 filament was kept under vacuum conditions when not in use and was preheated to 80 °C.

**Figure 4:**
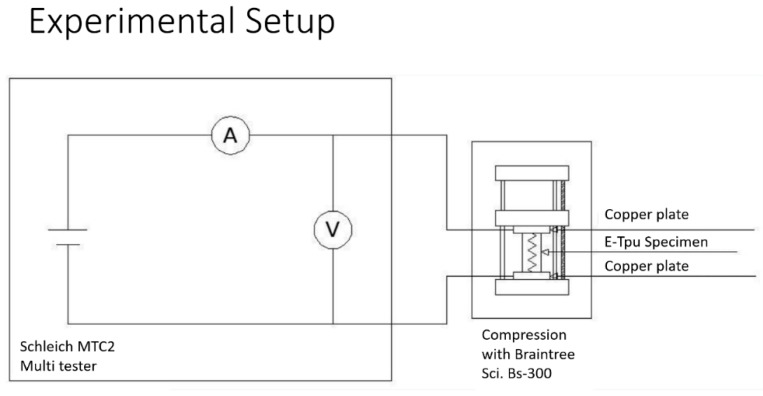
Experimental setup for the voltamperometric measurements.

The print core used was a 0.8 mm of diameter (type AA) while the Cura software was set up with a fast-type profile. The printing parameters are the TPU parameters recommended by the software, except for the following: (i) retraction was disabled, since enabling retraction is a variation in the filament output from the nozzle, which causes a cooling that prevents extrusion; (ii) ironing was enabled, since it makes the sample more compact and less prone to deformation horizontally during the printing process; (iii) the infill density was 100%, yet this value was reduced to 50% before bad filament deposition was experienced; (iv) the flow was 70%, which was found to be the optimal value under those conditions; and (v) the build plate temperature was 50 °C, since it was the lowest temperature that prevents detachment. Hence, the possibility of varying the process parameters was very low due to the limited processability of the filament on the Ultimaker. After a trial and error phase for finding a correct setup, the process parameters were set at a temperature of 220 °C, a material flow equal to 70% and a print speed equal to 30 mm/s. To evaluate the repeatability, each geometry was reproduced in 9 specimens, while each specimen was subjected to three compression cycles using a syringe pump: BRAINTREE SCIENTIFIC, INC. (Braintree, MA, USA) Mod. BS-300, from 0 to 2 mm, with a 0.25 mm step. At the end of each step, a Volt-amperometric measurement was carried out using the Multi-Purpose Tester: SCHLEICH (Schwäbisch Gmünd, Germany) Mod. MTC2, Resistance Range: 1 µΩ–100 KΩ, Test Voltage: 110 Vac, Frequency Test: 50 Hz. The experimental setup is shown in Figure 4.

At the start of the test, the TPU specimen was placed on the BRAINTREE SCIENTIFIC, INC. BS-300 Syringe pump in a horizontal position, with the two bases in contact with the copper plates (electrodes). Then, a voltamperometric approach has been used, applying a 110 V AC voltage (50 Hz) between the two electrodes, reading the measured resistance value on the Multi-Purpose Tester SCHLEICH MTC2 and switching off the power supply.

About half a minute after, the sample was compressed by means of a syringe pump, with 0.25 mm steps, up to 2 mm, using a voltamperometric method: apply a 110 V AC voltage, read the measured resistance value on the display and switch off the power supply.

This test has been carried out on the same sample three times on different days, so that possible hysteresis effects can be evaluated.

The same methodology has been applied to different kinds of specimens.

## 3. Results and Discussion

**Figure 5:**
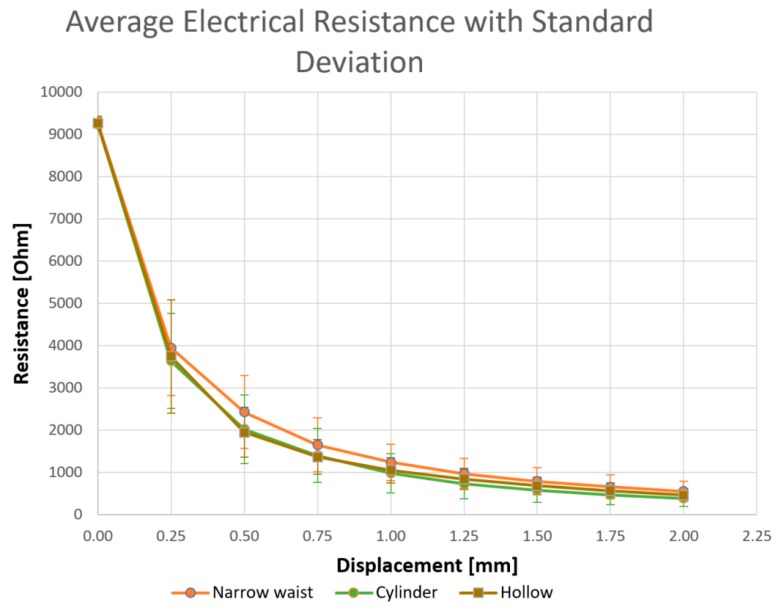
Measured electrical resistance and standard deviation of the different configurations of 3D-printed sensors.

The results of the tests are shown in Figure 5, where the measured electrical resistance of the different configurations of 3D-printed sensors is shown, together with the standard deviation.

Each value of resistance in the graph, for each value of displacement, is the average of 27 tests, with three repetitions for each of the nine specimens fabricated with that specific geometry.

The lowest value of the average resistance is measured on a solid cylinder workpiece, with a higher average section, but very similar values are measured on the hollow geometry, though they are characterized by a lower average section value. This behavior can be attributed to the skin effect, which is equal to the external surfaces of both these configurations. In fact, the skin effect is the tendency of the alternating current (AC) to distribute within a conductor in such a way that the current density grows, from the axis to the external surface of the conductor. This is consistent with the behavior of the measure on the narrow waist structure, where a higher average resistance is measured, which is higher than the surface and longer than the path of the current.

Another consideration is that the first measure, at 0.2 mm of displacement, is more repeatable (standard deviation is lower than 200) than the second one, which shows a very high standard deviation (higher than 1000) at 0.4 mm of displacement for all the configurations. The subsequent displacements show a reduction of the standard deviation, together with that of the resistance. The resistance decreases as the section of the specimen increases during upsetting, and the standard deviation is lowered, probably due to the stabilization of the manufacturing defects inside the specimen. The full specimens became less repeatable than the hollow ones, probably indicating higher and more repeatable variations on the external surface during the deformation. The most repeatable configuration for the considered experimentation was the hollow one, which showed the lowest standard deviation. These aspects must be further investigated.

Several issues have been faced during the manufacture of sensors, mainly due to the presence of carbon black filler in the TPU matrix. This addition tends to make the filament more brittle than standard TPU and leads to a deposition that is affected by discontinuities over the deposited beads.

Authors should discuss the results and determine how they can be interpreted from the perspective of previous studies and the working hypotheses. The findings and their implications should be discussed in the broadest context possible. Future research directions should also be highlighted.

## 4. Conclusions

The commercial carbon-filled TPU, analyzed in the present paper, has proven to be a candidate material for the production of 3D-printed displacement sensors. However, some limitations in fabricating the transducers from a 2.85 mm filament using Ultimaker 3 were experienced, and comparisons with 1.75 mm filaments and alternative machines should be conducted.

Moreover, further research on the low repeatability at low displacements and the higher performance of the hollow structure, in terms of repeatability, must be carried out.

Repeating the same tests on larger-size specimens to investigate a possible reduction of the effect of the manufacturing defects on repeatability, as well as on varying the frequency of the applied source, would also be interesting. In fact, higher frequencies should enhance the skin effect and confirm the authors’ hypotheses.

## References

[1] Ni Y., Ji R., Long K., Bu T., Chen K., Zhuang S. (2017). A review of 3D-printed sensors. Appl. Spectrosc. Rev..

[2] Flowers P.F., Reyes C., Ye S., Kim M.J., Wiley B.J. (2017). 3D printing electronic components and circuits with conductive thermoplastic filament. Addit. Manuf..

[3] Li K., Wei H., Liu W., Meng H., Zhang P., Yan C. (2018). 3D printed stretchable capacitive sensors for highly sensitive tactile and electrochemical sensing. Nanotechnology.

[4] Burstyn J., Fellion N., Strohmeier P., Vertegaal R. (2015). Printput: Resistive and capacitive input widgets for interactive 3D prints. Lect. Notes Comput. Sci..

[5] Leigh S.J., Bradley R.J., Purssell C.P., Billson D.R., Hutchins D.A. (2012). A Simple, Low-Cost Conductive Composite Material for 3D Printing of Electronic Sensors. PLoS ONE.

[6] MacDonald E., Wicker R. (2016). Multiprocess 3D printing for increasing component functionality. Science.

[7] Guo S.-Z.Z., Qiu K., Meng F., Park S.H., McAlpine M.C. (2017). 3D Printed Stretchable Tactile Sensors. Adv. Mater..

[8] Muth J.T., Vogt D.M., Truby R.L., Mengüç Y., Kolesky D.B., Wood R.J., Lewis J.A. (2014). Embedded 3D printing of strain sensors within highly stretchable elastomers. Adv. Mater..

[9] Xu Y., Wu X., Guo X., Kong B., Zhang M., Qian X., Mi S., Sun W. (2017). The Boom in 3D-Printed Sensor Technology. Sensors.

